# Strontium- and Copper-Doped Ceramic Granules in Bone Regeneration-Associated Cellular Processes

**DOI:** 10.3390/jfb15110352

**Published:** 2024-11-20

**Authors:** Yuliya Safarova (Yantsen), Assem Nessipbekova, Aizhan Syzdykova, Farkhad Olzhayev, Bauyrzhan Umbayev, Aliya Kassenova, Inna V. Fadeeva, Sholpan Askarova, Julietta V. Rau

**Affiliations:** 1 National Laboratory Astana, Nazarbayev University, Kabanbay batyr Ave. 53, 010000 Astana, Kazakhstan; assem.nessipbekova@nu.edu.kz (A.N.); syzdykova@nu.edu.kz (A.S.); folzhayev@nu.edu.kz (F.O.); bauyrzhan.umbayev@nu.edu.kz (B.U.); aliya.kassenova@nu.edu.kz (A.K.); shaskarova@nu.edu.kz (S.A.); 2 A. A. Baikov Institute of Metallurgy and Material Science RAS, Leninskie, 49, 119334 Moscow, Russia; fadeeva_inna@mail.ru; 3 Instituto di Struttura della Materia, Consiglio Nazionale delle Ricerche, ISM-CNR, Via del Fosso del Cavaliere 100, 00133 Rome, Italy; giulietta.rau@ism.cnr.it; 4 Department of Analytical, Physical and Colloid Chemistry, Institute of Pharmacy, I.M. Sechenov First Moscow State Medical University, Trubetskaya 8, Build. 2, 119048 Moscow, Russia

**Keywords:** tricalcium phosphate granules, strontium-doped tricalcium phosphate, copper-doped tricalcium phosphate, double-doped tricalcium phosphate, mesenchymal stem cells

## Abstract

Background: Pathological bone fracturing is an escalating problem driven by increasing aging and obesity. Bioceramics, particularly tricalcium-phosphate-based materials (TCP), are renowned for their exceptional biocompatibility, osteoconductivity, and ability to promote biomineralization. In the present study, we designed and characterized TCP porous granules doped with strontium (Sr) and copper (Cu) (CuSr TCP). Sr^2+^ ions were selected as Sr plays a crucial role in early bone formation, osteogenesis, and angiogenesis; Cu^2+^ ions possess antibacterial properties. Materials: The synthesized CuSr TCP granules were characterized by X-ray diffraction. Cytotoxicity and cell proliferation analyses’ assays were performed through the lactate dehydrogenase (LDH) activity and CCK-8 viability tests in rat bone marrow-derived mesenchymal stem cells (BM-MSCs). Hemolytic activity was carried out with human red blood cells (RBCs). Early and late osteogenesis were assessed with alkaline phosphatase (ALP) and Alizarin Red S activity in human osteoblast progenitor cells and rat BM-MSCs. The influence of CuSr TCP on angiogenesis was investigated in human umbilical vein endothelial cells (HUVECs). Results: We have demonstrated that media enriched with CuSr TCP in concentrations ranging from 0.1 mg/mL to 1 mg/mL were not cytotoxic and did not significantly affect cell proliferation rate motility. Moreover, a concentration of 0.5 mg/mL showed a 2.5-fold increase in the migration potential of BM-MSCs. We also found that CuSr TCP-enriched media slightly increased early osteogenesis. We also found that Sr and Cu substitutions in TCP particles significantly enhanced the measured angiogenic parameters compared to control and unsubstituted TCP granules. Conclusion: Our results demonstrate that TCP porous granules doped with Sr and Cu are biocompatible, promote osteodifferentiation and angiogenesis, and could be recommended for further in vivo studies.

## 1. Introduction

Bone fractures are a significant worldwide public health issue that imposes serious economic and social burdens [1]. Fractures can lead to absence from work, decreased productivity, disability, poor quality of life, loss of health, and high healthcare costs, imposing a substantial burden on individuals, their family members, and healthcare systems [2]. According to Ai-Min Wu et al., in 2019, there were 178 million new fractures, 455 million prevalent cases of acute or long-term fracture symptoms, and 25.8 million YLDs (years lived with disability) worldwide [3]. From a meta-analysis of 113 studies, the collective spending for hip fracture management in hospitals is estimated to be USD 10,075, and total health and social care costs per hip fracture averaged USD 43,669.7 at 12 months [4]. Given the global trend of increasing aging and obesity in the population and the associated increased risk of fractures, the search for new effective treatments for fractures is of great importance [5,6,7]. Particular attention should be paid to complex clinical conditions that require enhanced bone tissue regeneration, such as the reconstruction of large bone defects resulting from trauma, infection, tumor resection, and skeletal abnormalities, as they often cannot heal on their own [8].

Currently, there are many different approaches to restoring the impaired or “insufficient” process of bone tissue regeneration, among which the most frequently used is bone grafting—a surgical method in which the missing bone is replaced with a bone graft [9]. In bone grafting, the implantation of bone autografts, allografts, metal devices, porous glasses, and ceramics is used [10]. Each year, several million bone-grafting operations are performed worldwide, where the main bone grafts are natural bone autografts and allografts [11]. However, natural bone grafts have their own disadvantages and limitations, the main ones being their high cost, limited availability, and possible risks of infection of the recipient [12]. Therefore, an alternative approach is the use of synthetic bone grafts, which have no risk of disease transmission and good reproducibility of the chemical composition and porous structure [9]. In particular, biodegradable synthetic transplants are of considerable interest since they are capable of being absorbed into the human body and, over time, replaced by natural bone tissue, which makes it possible to avoid repeated surgical interventions.

Among the bioactive synthetic bone grafts available, the most commonly used are calcium phosphate ceramic implants: synthetic hydroxyapatite (Ca_10_(PO_4_)_6_(OH)_2_, HA) and tricalcium phosphate (Ca_3_(PO_4_)_2_, β-TCP) and their mixtures called biphasic calcium phosphates (BCPs) [13]. β-TCP is highly biocompatible and bioresorbable and enhances bone regeneration [14,15,16,17] due to the similarity in crystal structure and chemical composition with the inorganic phase of natural bone [18,19]. β-TCP is capable of filling critical-sized bone defects and possesses osteoinductive and osteoconductive properties, as well as the ability to promote biomineralization [19]. In addition, β-TCP is a more rapidly absorbable ceramic compared to HA, which makes β-TCP more convenient for clinical use [20]. It was shown that β-TCP grafts are completely replaced by natural bone within 3 years after transplantation [21].

Currently, β-TCP is commercially available and is used as a bone graft by brands such as ChronOS^®^, GUIDOR^®^ easy-graft, Poresorbs-TCP^®^, etc. Its efficacy and safety were proven in many clinical studies [18,22]. However, despite the faster bone regeneration compared to HA, the osteoinductive potential of β-TCP is low to achieve parameters identical to natural bone or bone grafts, and its resorption rate is still lower than the rate of new bone formation [19,23]. The introduction of transition metal ions, also present in the human body in trace amounts, can circumvent these problems by offering characteristics that are suited for specific applications. These ions can impart additional properties, such as enhanced mechanical strength and antimicrobial activity, to the ceramic material [24,25]. Moreover, the controlled release of these ions can stimulate cellular responses and promote tissue regeneration in biological environments [19,26,27].

Among the known trace elements, special attention is given to strontium, which is often used to improve the physicochemical properties of biomaterials [28]. It was shown that the replacement of calcium (Ca^2+^) by strontium (Sr^2+^) in the crystal structures of β-TCP increased its solubility and improved the mechanical properties of β-TCP, enhancing compressive strength and density [29]. It also enhanced its biocompatibility, promoting cell attachment, proliferation, and differentiation [30,31,32,33]. Sr-doped β-TCP exhibited better osteogenesis and slower degradation in vivo compared to pure β-TCP [30]. Recently, other studies have shown that β-TCP Si/Zn-substituted with Sr–apatite coating improved the osteoinductive properties of the graft [34]. The addition of Sr to collagen/β-TCP scaffolds increased alkaline phosphatase activity in mesenchymal stem cells [35]. Overall, Sr doping of β-TCP showed potential for enhancing bone regeneration and creating controlled-release bone graft materials [36].

Another important challenge in bone grafting with β-TCP implants is the lack of inherent antibacterial activity, which increases the risks of bacterial colonization and bone infection, potentially leading to serious complications in patients [37,38,39]. One promising approach to enhance the antibacterial properties of the implant is through chemical doping of bioceramics with ions that possess antibacterial characteristics [38,39]. Recently, elements such as silver (Ag), zinc (Zn), cerium (Ce), samarium (Sm), and copper (Cu) have been incorporated into implant coatings, effectively suppressing microbial growth at the implantation site [38,39]. In 2016, it was demonstrated that the antimicrobial activity of β-TCP-substituted compounds increased in the order of Ag < Fe < Cu < Zn [40]. Notably, there has been renewed interest in copper as an essential trace element with numerous biological functions, including antibacterial properties, angiogenesis, and osteogenesis stimulation [10]. Fadeeva et al. [41] reported that the addition of Cu ions to β-TCP improved its antibacterial properties without compromising the biocompatibility, making it a more attractive option than pure β-TCP for clinical applications.

However, given the fact that a higher copper content in biomaterials provides a better antibacterial effect, but increases cytotoxicity, a number of studies have utilized joint substitution or so-called binary doping of copper with various ions to reduce side effects [42,43,44], and it is reasonable to assume that strontium might be a good candidate for binary doping with Cu. As an example, Huang et al. [44] showed that binary doping with strontium and copper hydroxyapatite (SrCuHA) on commercially pure titanium (CP-Ti) effectively compensated for the cytotoxicity of Cu and stimulated osteogenic differentiation. In a similar study [43], a bioimplant composed of bioactive glass doped with strontium and copper had osteoinductive and antibacterial effects and also stimulated angiogenesis in vitro and promoted wound healing in rats. Lebedev et al. [25] showed that Sr, Cu-co-doped solid solutions of the Ca_9.5–x_Sr_x_Cu(PO_4_)_7_ composition possess significant inhibitory activity against pathogenic strains *Escherichia coli* and *Staphylococcus aureus*. Recent studies have demonstrated that Sr ions alone also possess an antimicrobial effect. Sr-TCP coatings, produced using the Ionized Jet Deposition technique, have significantly inhibited *Escherichia coli* and *Staphylococcus aureus* growth by reducing bacterial adhesion and biofilm formation [45]. Additionally, Rau et al. [46] demonstrated that a zinc–lithium (Zn-Li) biodegradable alloy coated with double-doped strontium and copper resorbable tricalcium phosphate (SrCu-TCP) using the Pulsed Laser Deposition method inhibited the growth of four bacterial strains by 24–35% [46].

Based on the information provided above, this study was focused on evaluating cytotoxicity, hemolytic properties, and osteogenic and angiogenic potentials in vitro of synthesized granules of tricalcium phosphate co-doped with strontium and copper, with the aim of progressing towards in vivo studies.

## 2. Materials and Methods

### 2.1. TCP Granule Synthesis and Doping with Sr and Cu

Double-substituted tricalcium phosphate powder with copper and strontium was synthesized using the solid-phase method, as previously described [25,47]. Briefly, the initial reagents were combined in a planetary mill in quantities determined by Reaction (1):

0.25Cu(NO_3_)_2_ + 0.25Sr(NO_3_)_2_ + 2CaHPO_4_⋅2H_2_O + 0.5CaCO_3_ →
Ca2.5Sr0.25Cu0.25(PO_4_)_2_ + 0.5CO_2_ + NO_2_ + 5H_2_O (1)

The mixture of 2.42 g of copper nitrate tetrahydrate, 2.12 g of strontium nitrate, 13.6 g of dicalcium phosphate dihydrate, and 5 g of calcium carbonate was placed in a corundum crucible and heated at 1150 °C in a chamber furnace with silicon carbide heaters for 4 h. The resulting ceramic was milled with corundum balls in a planetary mill for 30 min, after which the mixture was again placed in a corundum crucible and heated for 4 h. The described operations were repeated 5 times. The phase composition of the obtained powder was determined by an X-ray diffraction analysis using a Rigaku diffractometer (Tokyo, Japan) with Cu Kα radiation (λ = 0.154 nm) in the angular range of 10–60 degrees at a 2θ scale. The ceramic targets were obtained from synthesized powder of double-substituted tricalcium phosphate with copper and strontium by double-sided uniaxial pressing in steel molds at a specific pressing pressure of 200 kgf/cm^2^. The targets were sintered in a chamber furnace with silicon carbide heaters at a temperature of 1200 °C for 2 h. The solubility and ion release from the prepared ceramics were studied at 37 °C and pH 7.4 in a model solution of 0.9% sodium chloride solution with a TRIS buffer by measuring the concentration of calcium, copper, and strontium ions. The ratio of ceramics to a saline solution was 5 mg/mL. The ion concentrations were determined at specific time intervals in the model solution using an inductively coupled plasma atomic emission spectrometer, Ultima II (HORIBA Jobin Yvon SAS, Palaiseau, France).

Ceramic CuSr TCP granules were prepared as described in [48]. Briefly, a suspension of CuSr TCP powder with a 5% aqueous solution of polyacrylamide (PAA) was prepared in a powder: a PAA solution ratio of 1:5 by weight. The resulting suspension was impregnated into a polyurethane sponge, dried at room temperature, and then fired at a temperature of 1200 °C for 2 h. The phase composition of the obtained granules was determined as described above. The resulting size of the granules was in a range of 600–850 µm.

### 2.2. CCK-8 Viability Assay

CCK-8 (96992, Sigma Aldrich, St. Louis, MO, USA) was used to evaluate the proliferation of rat bone marrow mesenchymal stem cells (BM-MSCs) and human osteoblast cell line hFOB1.19 (CRL-3602, ATCC, Manassas, VA, USA) in vitro. In brief, 5 × 10^3^ cells were seeded into each well of a 96-well plate and incubated for 1, 2, and 3 days with CuSr TCP granule-enriched media in different concentrations—0.1, 0.5, and 1 mg/mL (3-day extract). Once incubated, 10 µL of the CCK-8 solution was added to each well and incubated for 4 h. A control group was incubated in the plain DMEM media with no implant extracts. Absorbance was measured at 450 nm using a microplate reader (Bio Tek Synergy H1, Winooski, VT, USA).

### 2.3. Lactate Dehydrogenase (LDH) Assay for Cytotoxicity

The 5 × 10^3^ BM-MSCs were seeded into each well and incubated for 24 h with granule-enriched (3-day extract) complete DMEM. The next day, a lysis buffer was added to the maximum LDH release wells, and sterile water to others. After 45 min of incubation at 37 °C, 50 µL media from each well was transferred to another plate. A 50 µL reaction mixture (C20300, Thermo Fischer Scientific, Waltham, MA, USA) was added to each well and incubated for 30 min at room temperature. After incubation, 50 µL of a stop solution was added to each well, and absorbance was read at 490 nm and 580 nm. No treatment wells were referred to as a spontaneous LDH activity group. The maximum LDH activity group served as a 100% cytotoxicity level. The cytotoxicity was calculated using the following equation:

(2) $$\%Cytotoxicity=\left[\frac{\left(Compound-treated\;LDH\;activity-Spontaneous\;LDH\;activity\right)}{\left(Maximum\;LDH\;activity-Spontaneous\;LDH\;activity\right)}\right]\times 100$$

### 2.4. Scanning Electron Microscopy (SEM)

CuSr TCP disks were seeded with 1 × 10^4^ BM-MSCs and cultured in complete DMEM (DMEM + 10% FBS (F2442, Sigma Aldrich, St. Louis, MO, USA) + 1% penicillin–streptomycin) for 48 h. The protocol for staining was adopted and modified by Geekiyanage et al. [49]. Then, cells were fixed with 2% paraformaldehyde for 10 min and stained with 1 M Osmium Tetroxide for 1 h, rinsed twice with Milli-Q water for 10 min, dehydrated with Ethanol (from 70% to 100%), and incubated with HDMS (hexamethyldisilazane) for 30 min. Following 30 min of drying, samples were covered with a 20 nm layer of gold. An assessment of the sample’s topography was performed by Zeiss Crossbeam 540 (Zeiss, Oberkochen, Germany).

### 2.5. Fluorescence Microscopy

CuSr TCP disks were seeded with 1 × 10^4^ BM-MSCs and cultured in complete DMEM (DMEM + 10% FBS (F2442, Sigma-Aldrich, St. Louis, MO, USA) + 1% penicillin–streptomycin) for 48 h. After the incubation period, cells were washed with phosphate-buffered saline (PBS), fixed in 4% paraformaldehyde for 30 min at room temperature, and permeabilized using 0.1% Triton X-100 for 4 min. Cells were then washed again with PBS and stained with Oregon Green 488 Phalloidin (O7466, Invitrogen, Waltham, MA, USA) for 1 h to visualize the actin cytoskeleton, followed by a 4 min counterstaining with DAPI to label nuclei. Fluorescence images were captured on a Cytation 5 Multimode Reader (Biotek, Winooski, VT, USA), using DAPI and GFP filter sets for DAPI and phalloidin visualization, respectively.

### 2.6. Transwell Migration Assay

BM-MSCs were seeded in the upper membrane of transwell plates with an 8 μm membrane pore size (141082, 12-well Carrier Plate with Cell Culture Inserts, Thermo Fischer Scientific, Waltham, MA, USA) with a concentration of 6 × 10^4^ cells each in a 100 μL serum-free DMEM (D6046, Sigma Aldrich, St. Louis, MO, USA) + 1% penicillin–streptomycin (15140122, Gibco, Waltham, MA, USA) medium per well and the lower wells were poured with 1 mL complete DMEM (DMEM + 10% FBS (F2442, Sigma Aldrich, St. Louis, MO, USA) + 1% penicillin–streptomycin). Then, the plate was incubated at 37 °C, 5% CO_2_, with a humidifier for 12 h. After incubation, the cells were fixed in 4% formaldehyde for 30 min and washed. Finally, 0.2% Crystal Violet (V5265, Sigma Aldrich, St. Louis, MO, USA) was used to fix and stain-migrate cells in the lower compartment of the upper chamber. Ultra-pure water was used to wash the stain, and then 5 pictures of each compartment were randomly photographed in a 200× microscope field. The total number of migrating BM MSCs was calculated and compared for each group using Image J 13.0.6 software. The methodology was modified from previously reported studies [50,51].

### 2.7. Alkaline Phosphatase Assay

Human premature osteoblast cells (hFOB1.19, CRL-3602, ATCC, Manassas, VA, USA) were seeded at a density of 8 × 10^4^ cells per well to a 48-well plate and cultured until 90% confluency was reached. Then, cells were transferred to 39 °C for osteogenic differentiation induction. Further, cells were treated with CuSr TCP granule-enriched media (3-day extract) for 2 weeks. Then, cell medium supernatants were collected, and alkaline phosphatase (ALP) levels were measured using an ALP Assay Kit (ab83369, Abcam, Cambridge, UK). An ALP enzyme of known concentration was plated in a serial dilution to create a standard curve. pNPP substrates were added to each standard and sample well and incubated for 60 min at room temperature. An ALP enzyme converts pNPP substrates to a colored p-nitrophenol (pNP). After incubation, a stop solution was added, and absorbance was recorded at OD405nm with a microplate reader (Bio Tek Synergy H1, Winooski, VT, USA). Groups with plain DMEM media served as a negative control for differentiation. No treatment group served as a control.

### 2.8. Alizarin Red S

BM-MSCs were seeded at 8 × 10^4^ cells per well density into the 48-well plate and cultured until 90% confluency. Then, the cells were cultured in osteogenic media (Osteogenesis Assay Kit, ECM815, Merck, Darmstadt, Germany) and incubated with granules for 3 days. At week 5 of differentiation, cells were washed, fixed with 500 µL 4% formaldehyde for 30 min, and stained with 500 µL 2% Alizarin Red S (A5533, Sigma Aldrich, St. Louis, MO, USA) for 1 h, then washed 5 times with MulliQ water for 30 min each time. After the washing, the stain was extracted from the differentiated monolayer with 400 μL 10% acetic acid, heated at 85 °C for 10 min, and centrifuged to 20,000 rcf for 15 min. The supernatant was collected, and the pH was adjusted to 4.1–4.5. Then, the supernatant was transferred to a microplate reader (OD: 405 nm), and the concentration of Alizarin Red S was calculated from the standard curve obtained from the standards.

### 2.9. Angiogenesis Assay

An angiogenesis starter kit (A14-609-0, Gibco, Waltham, MA, USA) was used to assess the angiogenesis. Human umbilical vein endothelial cells (HUVECs, C-003-5C, Gibco, Waltham, MA, USA) were pre-treated in media with different TCP granule concentrations (1 mg/mL, 0.5 mg/mL, 0.1 mg/mL) for 72 h. HUVECs in the concentration of 4.2 × 10^3^ per well were seeded in a GelTrex matrix-coated 24-well plate for 16 h. After 16 h, the plate was stained with 2 µg/mL of Calcein AM, incubated for 30 min, and imaged at 4× magnification with Cytation 5 (BioTek, Winooski, VT, USA). Images were assessed with the Image J 13.0.6 Angiogenesis Analyzer Plugin 1.0.

### 2.10. Blood Hemolysis Test

The in vitro hemolysis assay was performed on human erythrocytes. All procedures related to the blood collection were performed according to the protocols approved by the Local Ethics Committee of National Laboratory Astana (Registration number IORG 0006963, N02-2022, 1 April 2022). Briefly, blood samples were collected from healthy volunteers in K2-EDTA vacutainers and centrifuged at 500× *g* for 10 min. After several PBS washes, the plasma from the fresh blood sample was removed, and red blood cells (RBCs) were diluted to prepare a 4% (*v*/*v*) RBC solution in PBS. Subsequently, 800 μL of the RBC solution in separate Eppendorf microtubes was mixed with either 200 μL of PBS (used as a negative control), a Triton-X solution (positive control), or TCP granules diluted in 0.2 mL of PBS in concentrations of 0.1, 0.5, and 1 mg/mL. The mixture was then incubated for 2 h at 37 °C with mild stirring breaks every half-hour. Following the incubation period, the mixture was centrifuged for 10 min at 2000× *g*. Then, 100 μL of the supernatant was taken into the 96-well plates. The absorbance (OD) at 570 nm was determined using a Synergy Hybrid H1 Microplate Reader (Biotek, Winooski, VT, USA). The percentage of hemolysis was calculated using the following equation:

(3) $$\%\;hemolysis=\left[\left(At-Ac\right)/(A100\%-Ac)\right]\times 100$$

## 3. Results

### 3.1. Characterization of CuSr TCP

**Figure 1:**
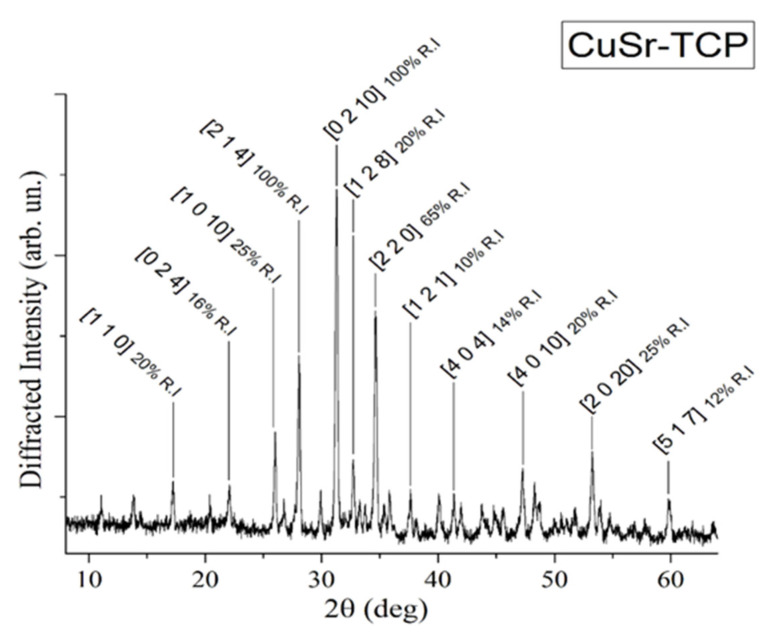
XRD pattern of CuSr TCP ceramic target.

The XRD pattern of the CuSr-substituted TCP ceramic is shown in Figure 1.

The analysis of the diffraction pattern indicated that the sample is of the β-TCP structural type, in agreement with the work of Deyneko et al. [47]. No diffraction peaks of impurities from apatite or pyrophosphate phases were detected, thereby verifying the completeness of the synthesis process. The influence of incorporated ions on structural properties of TCP was investigated and reported by us earlier in [45].

**Figure 2:**
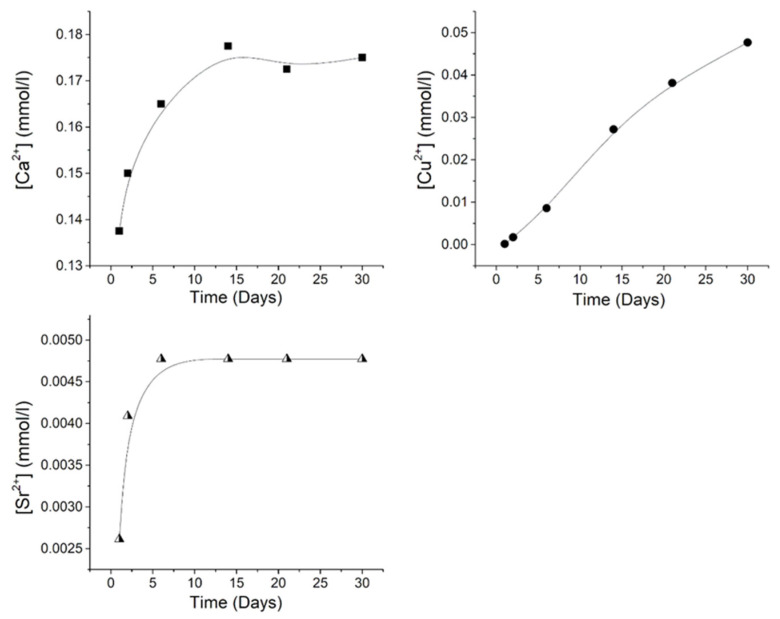
The cumulative release amount of Ca^2+^ (filled squares), Cu^2+^ (filled dots), and Sr^2+^ (half-filled triangles) ions from CuSr TCP ceramics after soaking in model liquid over 30 days. The release profile of each ion is plotted with a black line.

The cumulative release of Ca^2+,^ Cu^2+^, and Sr^2+^ ions from CuSr TCP ceramics after soaking for 30 days in a 0.9% sodium chloride solution with the TRIS buffer is illustrated in Figure 2. Despite the possible binding of Cu^2+^ ions with TRIS, before determining the Cu^2+^ release in the solution, we treated the solution containing TRIS and copper ions with hydrochloric acid, which breaks down the copper complexes [52]. As can be seen from the obtained results, distinct release behaviors are observed for all detected ions. The concentration of Ca^2+^ reaches a plateau of approximately 0.175 mmol/L after 15 days of soaking. Conversely, the Cu^2+^ concentration exhibits an almost linear trend throughout the entire 30-day period, with the highest value of 0.048 mmol/L, while the Sr^2+^ concentration plateaus after 7 days, reaching a value of 0.00475 mmol/L.

### 3.2. Cytotoxicity and Hemocompatibility of CuSr TCP

**Figure 3:**
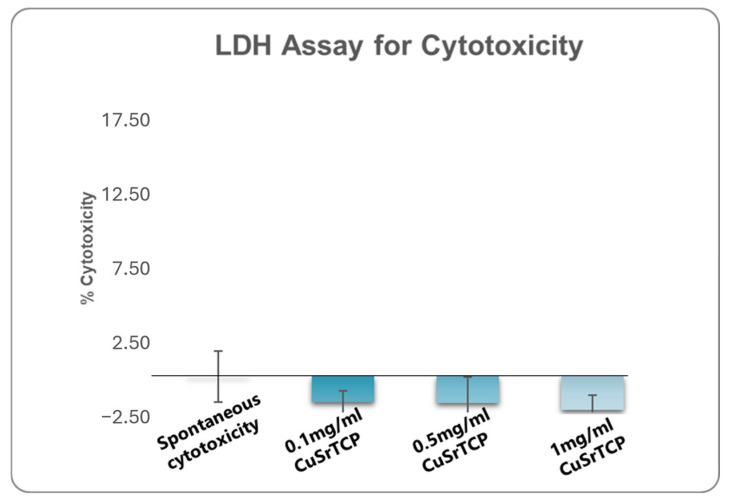
Lactate Dehydrogenase Assay for cytotoxicity. BM MSCs were cultured in CuSr TCP-enriched media (3-day extract) for 24 h. CyQUANT™ LDH Cytotoxicity Assay (C20300, Thermo Fischer Scientific, Waltham, MA, USA) was used to evaluate cytotoxicity.

One of the fundamental assays commonly employed in standard cytotoxicity testing is the assessment of lactate dehydrogenase (LDH) release. LDH is a stable cytoplasmic enzyme present in all cells, and its release into the extracellular environment serves as a marker of cell membrane integrity [53]. When cells undergo damage or death, primarily through necrosis or late apoptosis, the compromised cell membrane allows LDH to leak into the surrounding culture medium. The quantification of the amount of LDH provides a reliable measure of cell death and membrane damage. In our study, media enriched with Sr, Cu-TCP in concentrations ranging from 0.1 mg/mL to 1 mg/mL did not show significant cell damage that would exceed 2% (Figure 3).

**Figure 4:**
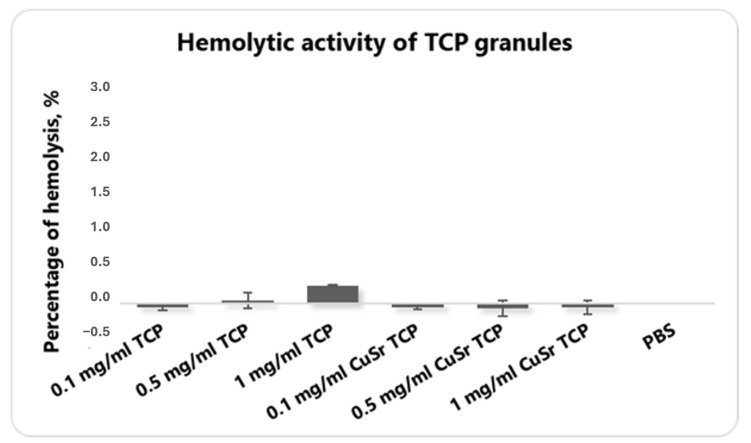
The blood hemolysis test with human RBCs. Blood samples were collected from healthy volunteers in K2-EDTA vacutainers, and plasma and buffy coats were removed. RBC solutions were incubated with various concentrations of CuSr TCP and TCP alone at 37 °C for 2 h. Absorbance was measured at 570 nm on a 96-well plate with Hybrid Reader Synergy H1 (Biotek, Winooski, VT, USA). The percentage of the hemolysis was calculated.

The hemolytic activity assay is an essential part of preclinical safety testing, ensuring that the material is biocompatible and reducing the risk of systemic toxicity during in vivo experimentation. Hemolysis refers to the destruction of red blood cells (RBCs), leading to the release of hemoglobin into the surrounding plasma. An in vitro hemolytic assay evaluates the potential for a material to cause RBC membrane damage, which is important for determining its biocompatibility and safety before proceeding with in vivo studies. A hemolysis rate of less than 5% is generally considered acceptable for materials intended for in vivo applications, as this indicates the minimal disruption of RBC integrity. None of the groups demonstrated hemolysis that exceeds even 1% (Figure 4). The PBS solution served as a control.

### 3.3. Effects of CuSr TCP on Cell Proliferation and Motility

**Figure 5:**
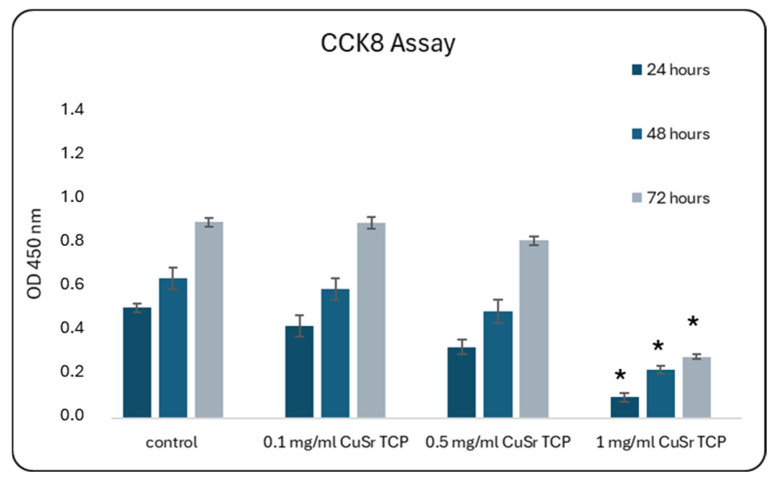
Proliferation Assay with CCK-8 during 72 h. Mesenchymal stem cells were cultured in CuSr TCP-enriched media (3-day extract) for 24–72 h. CCK-8 (96992, Sigma Aldrich, St. Louis, MO, USA) was used to assess number of proliferated cells. *—*p* value ≤ 0.05 compared to control.

The next step in accessing the in vitro biocompatibility of double-ion-doped CuSr-TCP was aimed at elaborating the concentration of ceramic granules that would not affect cell growth. We assessed the proliferation of primary rat MSCs as a model of the bone progenitor cells during 72 h using the CCK8 (Sigma-Aldrich) assay. CCK8 is based on producing a water-soluble formazan dye when the tetrazolium salt, WST-8, is reduced by dehydrogenases in live cells. In our study, media enriched with 0.1 mg/mL and 0.5 mg/mL of CuSr TCP did not significantly affect cell growth and were comparable to the no-treatment control (Figure 5).

**Figure 6:**
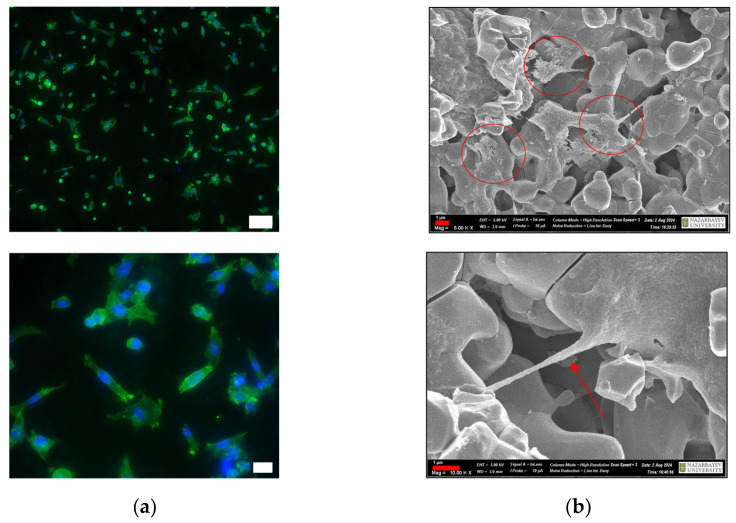
IFM images of BM-MSCs on the surface of CuSr TCP granules: (**a**) cells were seeded in a concentration of 1 × 10^4^ cells per well, fixed, and double-stained with DAPI and phalloidin. Imaged with Zeiss Crossbeam 540 (Zeiss, Oberkochen, Germany), and scale bars are 100 µm for the upper and 20 µm for the lower image. (**b**) SEM images of BM-MSCs on the surface of CuSr TCP granules. Cells were seeded in a concentration of 1 × 10^4^ cells per well, fixed, stained with 1 M Osmium Tetroxide, and covered with 20 nm gold. Scale bar: 1 µm.

Cells can sense the three-dimensional structure of the substrate on which they grow, and, in turn, substrate topography can significantly affect cellular morphology and functions. In our study, we applied inverted fluorescent and scanning electron microscopy (SEM) to assess the interaction of BM-MSCs with the CuSr TCP surface. Immunofluorescent images of the BM-MSCs attached to the CuSr TCP disks after 2 days of culture are presented in Figure 6a. Visualized through phalloidin staining, the actin filaments exhibited a well-defined structure with prominent stress fibers and cortical actin, indicating proper cytoskeletal organization. SEM images of the BM-MSCs attached to the CuSr TCP disks after 2 days of culture are presented in Figure 6b. We found that BM-MSCs are capable of adhering inside the pores (shown with arrows) while maintaining spindle fibroblast-like morphology typical for MSCs.

**Figure 7:**
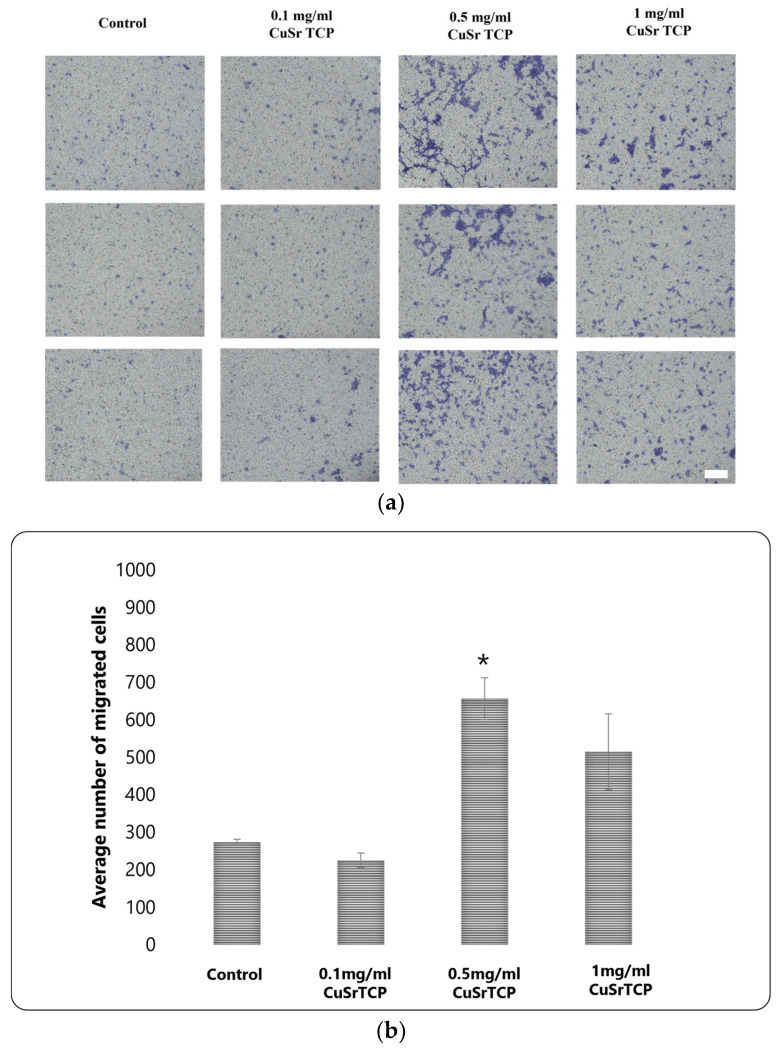
Transwell migration assay: (**a**) Images were analyzed with ImageJ and migrated cells were counted (scale: 100 µm); (**b**) Average number of migrated cells across the groups, *—*p* value ≤ 0.05 compared to control group.

A Transwell migration assay provides valuable insights into how cells respond to environmental cues. BM-MSCs were cultured in CuSr TCP-enriched media (3-day extract) for 72 h, then were seeded in a density of 6 × 10^4^ cells per well in 12-well plate inserts and allowed to migrate for 8 h, and then fixed, stained with Crystal Violet, and imaged under the microscope Axio Observer (Zeiss, Oberkochen, Germany) (Figure 7a). According to our data, media enriched with 0.1 mg/mL of granules did not significantly affect the migration ability of MSCs, while the cells in a group of 0.5 mg/mL CuSr TCP migrated more efficiently (2.5 times) than the control (Figure 7b). The excess ions released from the CuSr TCP granules could modify the biology of a cell, resulting in increased cell motility. Overall, it is a valuable tool in developing therapeutic strategies targeting cell migration and invasion.

### 3.4. Effects of CuSr TCP on the Osteogenic Potential of BM-MSCs

**Figure 8:**
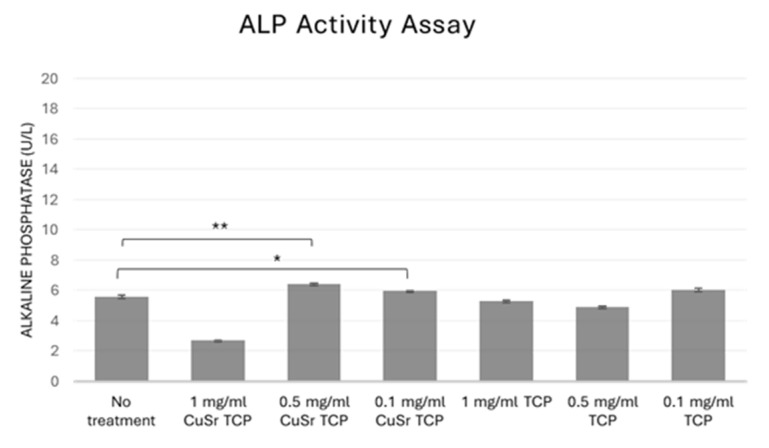
The alkaline phosphatase activity assay. hFOB1.19 cells were cultured in CuSr TCP- or TCP-enriched media (3-day extract) for 14 days. An Alkaline Phosphatase Assay Kit (ab83369, Abcam, Cambridge, UK) was used to assess the osteogenic differentiation. *—*p* value ≤ 0.05 compared to the no-treatment group, **—*p* value ≤ 0.005 compared to the no-treatment group.

In bone biology, alkaline phosphatase (ALP) is produced by osteoblasts. It is considered a key marker of bone formation, as it facilitates the deposition of calcium and phosphate in the bone matrix. Measuring ALP activity can, therefore, provide insights into bone metabolism, regeneration, and disorders like osteoporosis. In tissue engineering and regenerative medicine, assessing ALP activity is essential when developing biomaterials or therapies to promote bone growth or healing, as it is an early indicator of osteogenic differentiation. In our study, human osteoblast progenitor cells (hFOB1.19) were cultured in CuSr-TCP- or TCP-enriched osteogenic media for 14 days. According to the results shown in Figure 8, TCP-enriched media did not significantly affect the osteogenic differentiation of the human preosteoblasts. At the same time, a group treated with media enriched with 0.1 mg/mL and 0.5 mg/mL of CuSr TCP showed a 5–7% increase in early osteogenesis compared to the control, suggesting that Sr, Cu-TCP may stimulate osteogenic properties of bone progenitor cells.

**Figure 9:**
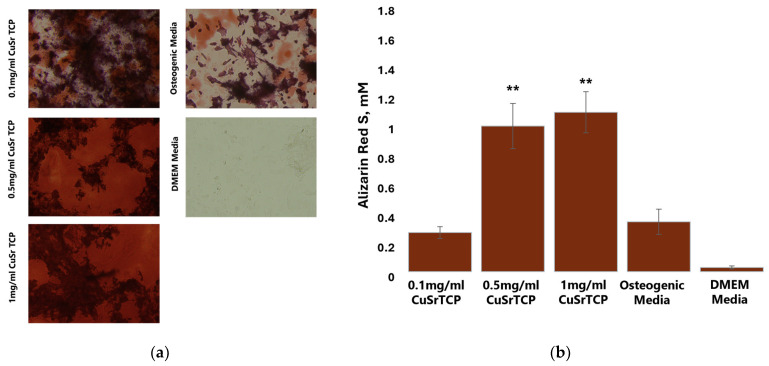
The Alizarin Red S Assay. BM-MSCs were cultured in CuSr TCP-enriched media (3-day extract) for 72 h. Alizarin Red S (A5533, Sigma Aldrich, St. Louis, MO, USA) was used to stain cells for osteogenic differentiation. (**a**) Microscopic images of osteogenic differentiation; (**b**) Quantitative analysis of osteogenic differentiation across the groups, **—*p* value ≤ 0.005 compared to the osteogenic media group (scale: 100 µm).

To further confirm the osteogenic potential of Sr, Cu-TCP, Alizarin Red S staining was performed on BM-MSCs in their fifth week of osteogenesis (Figure 9a), with the concentration of the dye in stained cell extracts being calculated according to the standard curve (Figure 9b). As Alizarin Red S dye stains calcium deposits in osteoblasts, this method is considered the main approach to quantify osteoblast mineralization and it is a marker of osteogenesis. To perform osteogenesis, osteoblast differentiation media were enriched with TCP ceramic porous granules modified with Sr and Cu at 0.1 mg/mL, 0.5 mg/mL, and 1 mg/mL. The results confirmed that CuSr TCP increased osteogenic activity and osteoblast mineralization in BM-MSCs in a concentration-dependent manner, with granules’ concentration of 1 and 0.5 mg/mL inducing the highest osteogenic activity as compared to undifferentiated cells and cells that have undergone standard differentiation with no CuSr TCP enrichment.

### 3.5. Angiogenic Potential of CuSr TCP

**Figure 10:**
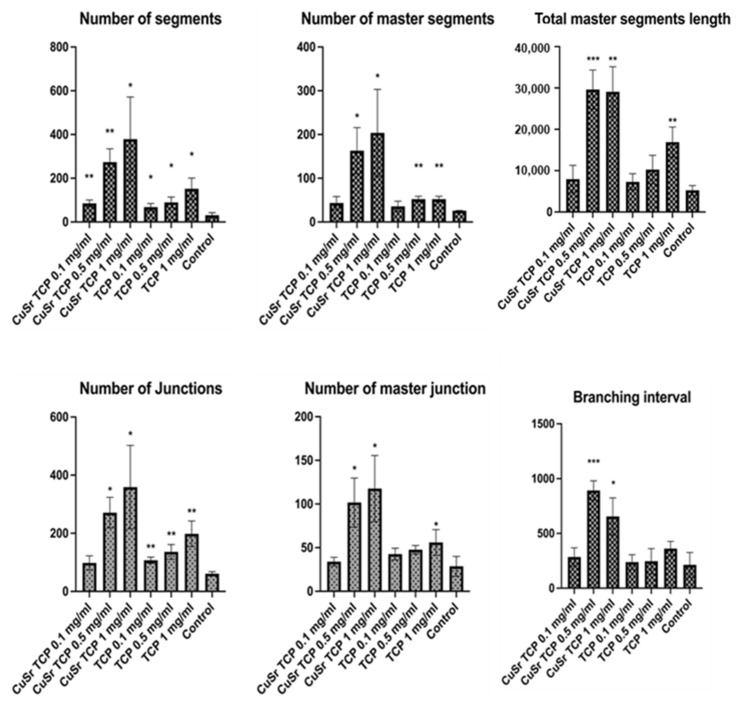
The angiogenesis assay. HUVECs (C-003-5C, Gibco, Waltham, MA, USA) were pre-treated in CuSr TCP-enriched media (3-day extract) for 72 h and seeded in the concentration of 4.2 × 10^3^ per well in a GelTrex matrix-coated 24-well plate for 16h. Cells were then stained with Calcein AM and imaged at 4X magnification. Images were assessed with the Image J Angiogenesis Analyzer Plugin [54]. *—*p* value ≤ 0.05 compared to the control group; **—*p* value ≤ 0.005 compared to the control group; ***—*p* value ≤ 0.0005 compared to the control group.

**Figure 11:**
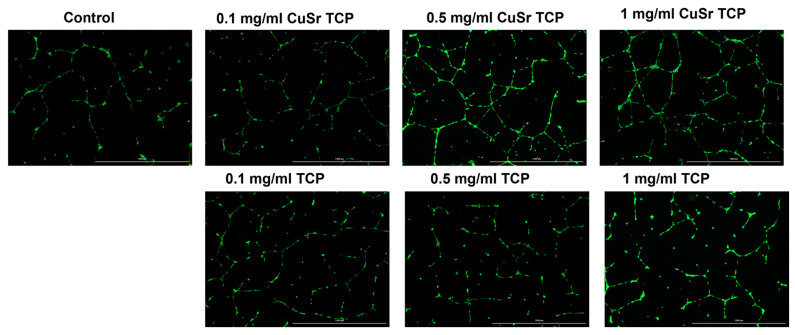
Representative images of angiogenesis assay. Images were acquired with Cytation 5 Multimode Reader (Biotek, Winooski, VT, USA) (scale: 1000 µm).

The influence of Sr- and Cu-substituted TCP on angiogenesis was investigated in human umbilical vein endothelial cells (HUVECs). Key parameters examined included the number of junctions, branches, master junctions, segments, master segments, total branching length, total branch length, branching interval, and total master segment length (Figure 10). Media enriched with CuSr TCP in concentrations of 0.5 mg/mL and 1 mg/mL showed the most prominent angiogenic effect. The number of segments in those groups was 8 and 12 times higher, respectively. The number of master segments and following segments’ length were subsequently increased. During the vessel development, cells form tubular structures and begin sprouting. The number of junctions is considered to be a measure of the sprouting. Both groups (0.5 and 1 mg/mL) showed a 4.5- and 6-fold increase in junctions, master junctions, and the branching interval compared to the control. The TCP-treated groups, while effective to some extent, do not reach the same level of effect as CuSr-treated groups. The results demonstrated that Sr and Cu substitutions in TCP particles significantly enhanced the measured angiogenic parameters compared to control and unsubstituted TCP granules. Furthermore, the angiogenic effects were concentration-dependent, with 0.5 mg/mL Cu-Sr-TCP showing better results than 0.1 mg/mL, and 1 mg/mL showing the most substantial improvements. Representative images were taken for each group to demonstrate the tube-like formations and sprouting (Figure 11).

## 4. Discussion

Tricalcium phosphate ceramics offer significant advantages in bone engineering due to their biocompatibility, biodegradability, and osteoconductivity. Unlike hydroxyapatite, TCP is resorbable and can be completely replaced by new bone tissue [55]. TCP ceramics promote rapid bone infiltration and ingrowth when implanted in cancellous or cortical bone. They can be fabricated into porous scaffolds with suitable mechanical properties and porosity for bone tissue engineering [56]. TCP ceramics have shown superior tissue compatibility compared to other synthetic materials and have been successfully used in various clinical applications, including periodontal defect repair.

We previously reported the antimicrobial properties of double-substituted CuSr TCP powder, revealing a significant inhibition of microbial growth [47]. Specifically, CuSr TCP powder demonstrated a remarkable growth inhibition, reaching 92.0%, 95.5%, 64.9%, 96.3%, and 70.9% against *Staphylococcus aureus*, *Pseudomonas aeruginosa*, *Escherichia coli*, and *Enterococcus faecalis* bacteria strains and *Candida albicans* fungus, respectively, highlighting its potent effectiveness across a range of pathogenic microorganisms [47]. In the present study, we have assessed cytotoxicity, hemolytic properties, and osteogenic and angiogenic potentials of the CuSr TCP granules. During the studies, none of the concentrations revealed an adverse effect on cell viability that would exceed spontaneous cell death events. Another essential preliminary step in evaluating the biocompatibility of materials is an analysis of the hemolytic activity of TCP granules. This assay assesses the potential of the material to cause the destruction of red blood cells, which can lead to adverse biological responses if the material is incorporated into the body. The results from the hemolytic activity were promising, showing the hemolytic activity in a range of less than 2%, indicating that CuSr TCP granules exhibit low hemolytic potential.

The optimal concentration for cell growth in CuSr TCP-enriched media was also established during the proliferation studies, with the highest dose of 1 mg/mL reducing the cells’ ability to proliferate. BM-MSCs were also seeded directly on the surface of CuSr TCP granules. Immunofluorescent images have demonstrated that the cells maintained a healthy morphology, as evidenced by the organized and intact arrangement of F-actin filaments within the cytoskeleton. This arrangement is crucial for cell integrity, shape maintenance, and overall cellular health, suggesting that the cells were not only alive but also functionally active and structurally intact. SEM images confirmed the spindle-shaped fibroblast-like morphology typical for MSCs, along with their migratory behavior, as we observed the cells adhering within the pores.

Further, the motility potential of BM-MSCs cultured in CuSr TCP-enriched media was assessed with a Transwell migration assay. According to our data, media enriched with 0.1 mg/mL of granules did not significantly affect the migration ability of MSCs, while the cells in a group of 0.5 mg/mL CuSr TCP migrated more efficiently than the control. This enhanced migratory response at the 0.5 mg/mL concentration is likely attributable to the elevated release of copper and strontium ions from CuSr TCP, which may influence pathways associated with cell motility, such as adhesion and cytoskeletal rearrangement. Some articles suggest that copper ions can stimulate the migration of stem cells through the upregulation of hypoxia-inducible factor 1α (HIF-1a) and the rho family GTPase 3 (Rnd3) [57]. Higher ionic release of metals at a concentration of 1 mg/mL, however, could limit the migratory capabilities of the cell, similarly to how a much lower proliferation was observed of mesenchymal stem cells incubated at 1 mg/mL during the CCK8 assay. These findings highlight the potential of CuSr TCP to optimize cell migration, suggesting that specific concentrations could be strategically applied in developing therapeutic approaches that target cell migration. The findings of He et al. [58] indicated that the improved migration potential of MSCs promoted osteogenic differentiation by activating the canonical Wnt-β-catenin pathway. In this context, the positive effect of CuSr TCP on the migration of cells would favor the process of osteogenesis.

Further confirming the osteogenic potential of new Sr- and Cu-TCP, early and late osteogenesis analyses demonstrated a significant positive effect in the group treated with TCP doped with Sr and Cu. In this regard, Sr^2+^ ions have been shown to stimulate osteogenic differentiation [59] and suppress osteoclast activity [60,61]. One of the mechanisms by which Sr^2+^ can influence cellular functions is through its involvement in calcium homeostasis [62], specifically by binding to the calcium-sensing receptor (CaSR) homolog [63]. This receptor normally senses the levels of extracellular Ca^2+^ ions but also can be activated by divalent Sr ions and, therefore, activate downstream signaling pathways [59], leading to the enhanced proliferation and differentiation of osteoblasts, stimulating the expression of osteogenic genes such as osteocalcin and bone morphogenetic protein-2 (BMP-2), which are crucial for bone formation, growth, and mineralization in the process of osteogenesis [56]. Further, the activation of Runt-related transcription factor 2 (RUNX2) takes place. RUNX2 is a key transcription factor in the matrix mineralization and differentiation of osteoblasts, and its activation induces the expression of bone differentiation markers such as alkaline phosphatase and osteocalcin [60,64,65]. In contrast, osteoclastogenesis refers to the process of osteoclast differentiation, the cells primarily responsible for bone resorption. Sr^2+^ ions have been shown to induce apoptosis of mature osteoclasts and decrease the receptor activator of NFkB ligand (RANKL) mRNA content [60,61]. Additionally, β-TCP ceramics themselves can stimulate the expression of bone-related genes and proteins, including BMP-2, TGF-β, and Runx2 [66].

While strontium plays a well-documented role in promoting osteogenesis and inhibiting osteoclast activity, copper is equally significant in bone tissue engineering due to its influence on osteoblast differentiation and angiogenesis. Low concentrations of Cu^2+^ (0.01–1 wt%) in TCP and calcium phosphate cement (CPC) have been shown to enhance osteoblast differentiation, collagen synthesis, and the upregulation of osteogenic and angiogenic genes [67,68,69]. Similarly, Cu-doped bioactive glass scaffolds have demonstrated promising results by promoting bone regeneration and blood vessel formation in vivo, highlighting copper’s role in both osteogenesis and vascularization [70,71]. However, it is essential to control copper concentrations carefully, as higher levels (>2 wt%) can become cytotoxic and inhibit bone formation [72]. Furthermore, studies on the combination of copper with other bioactive elements like zinc and silicon in calcium phosphate scaffolds have demonstrated synergistic effects, enhancing both osteogenic and angiogenic outcomes [69,73]. These findings underscore the importance of a controlled Cu^2+^ release from bone graft materials, suggesting that copper, alongside strontium, offers a promising strategy for improving bone regeneration and vascularization when incorporated in an appropriate concentration.

Angiogenesis plays a vital role in bone regeneration, as the formation of new blood vessels is essential for delivering nutrients and oxygen to the regenerating tissue. It has been shown that β-TCP can promote angiogenesis through increased VEGF secretion [74,75]. In our study, an in vitro analysis of angiogenesis was performed with human umbilical vessel endothelial cells pre-treated in CuSr TCP-enriched media. These cells were chosen based on their ability to form tubular vessel-like structures. Further, the walls of the newly formed vessels were stained with a fluorescent dye and imaged, and a number of parameters were analyzed. We demonstrated that media enriched with CuSr TCP in concentrations of 0.5 mg/mL and 1 mg/mL significantly increased the number of segments, the number of master segments, the total length of master segments, the number of junctions and master junctions, and the branching interval. The number of segments refers to the individual sections between two junctions. In contrast, master segments are the main or primary vessels in the network from which smaller branches or secondary segments originate [54]. An increase in the number of master segments indicates a more complex and hierarchical vascular network, which is crucial for the stability and functionality of newly formed vessels. The total length of master segments implies the development of longer, potentially more durable vessel-like structures. Our data suggest that CuSr TCP granules not only enhance the quantity but also improve the quality of the vascular structures, providing a solid foundation for the overall vessel network.

The enhanced angiogenesis seen in HUVECs suggests that Sr- and Cu-doped TCP granules may promote vascularization in bone repair sites, which is critical for successful bone regeneration. Increased blood vessel formation can improve the supply of nutrients, oxygen, and signaling molecules to the affected area, supporting both the survival and differentiation of osteogenic cells. This vascular support is particularly valuable in compromised bone environments, such as osteoporotic or large bone defects, where healing is often delayed. Thus, the pro-angiogenic effect observed in our study highlights the potential of CuSr TCP granules not only as an osteogenic but also as an angiogenic biomaterial, which could improve outcomes in clinical applications requiring robust vascularization for bone healing.

However, our study has some limitations. One such limitation is the concentration range tested, as we focused on a narrow set of doses to establish baseline effects. Future studies with a broader concentration range could more effectively capture dose-dependent responses. Additionally, while our study assessed osteogenic and angiogenic markers, it did not include the direct assessments of specific signaling pathways activated by Sr and Cu, which could yield deeper insights into their mechanistic roles. In addition, in vivo testing could offer a more comprehensive understanding of the material’s behavior in a physiological environment and will be a key focus of our future studies.

## 5. Conclusions

Our findings demonstrate that TCP granules double-doped with Cu and Sr ions exhibit significant potential for promoting osteodifferentiation in vitro, with minimal cytotoxic and hemolytic effects, underscoring their biocompatibility—an essential quality for medical applications. The material also showed promising results in both early and late osteogenesis, evidenced by increased ALP activity and Alizarin Red S staining, highlighting its positive impact on bone formation markers. Additionally, an angiogenic analysis in HUVECs revealed substantial improvements in vascular formation metrics, suggesting that CuSr-doped TCP could facilitate both bone regeneration and vascularization, crucial factors for effective bone healing and integration. However, additional in vivo research will be essential to validate these results, providing deeper insights into the material’s long-term safety, efficacy, and clinical potential in bone regeneration applications.

## Data Availability

The original contributions presented in the study are included in the article, further inquiries can be directed to the corresponding author.
